# Optimization of process parameter for green die sinking electrical discharge machining: a novel hybrid decision-making approach

**DOI:** 10.1038/s41598-025-92713-2

**Published:** 2025-04-18

**Authors:** Jagadish, Divya Zindani, Arun Selvam, Ghanshyam G. Tejani, A. Johnson Santhosh

**Affiliations:** 1 Indian Statistical Institute, Bangalore, India; 2 Sri Sivasubramaniya Nadar (SSN) College of Engineering, Kalavakkam, Tamil Nadu India; 3 Department of Mechanical Engineering, Sri Sivasubramaniya Nadar (SSN) College of Engineering, Kalavakkam, 603 110 India; 4 Department of Research Analytics, Saveetha Dental College and Hospitals, Saveetha Institute of Medical and Technical Sciences, Saveetha University, Chennai, 600077 India; 5 Department of Industrial Engineering and Management, Yuan Ze University, Taoyuan City, 320315 Taiwan; 6 Faculty of Mechanical Engineering, Jimma Institute of Technology, Jimma University, Jimma, Ethiopia

**Keywords:** TODIM, Green manufacturing, Process parameters, Single valued neutrosophic sets, Electrical discharge machining, MCDM, Electrical and electronic engineering, Applied mathematics

## Abstract

Electrical discharge machining (EDM) generates toxic emissions and hazardous waste, posing significant health risks for operators and environmental concerns. Aligning with the UN Sustainable Development Goals (SDGs) for Good Health & Well-being (SDG 3) and Responsible Consumption & Production (SDG 12), it is crucial to optimize EDM to reduce its environmental impact. This study introduces a Decision Support System (DSS) that uses a new approach to prospect theory based on exponential-logarithmic single-valued neutrosophic sets (± Log-SVNS) to find the best EDM parameters for “green” die-sinking EDM. Taguchi orthogonal array has been used to design the EDM experiments considering different levels of following process parameters: peak current, pulse duration, dielectric level, and flushing pressure. Log-SVNS structures expert evaluations on output responses which includes process time, tool wear ratio, energy consumption, aerosol concentration, and dielectric usage, and aggregates them with hybrid averaging and geometric operators. The proposed method employs prospect theory to finally determine the optimal machining parameters. The best performance is seen when the peak current is 2 A, the pulse duration is 520 µs, the dielectric level is 80 mm, and the flushing pressure is 0.5 kg/cm². This outcome is based on the µLog-SVNS hybrid average TODIM (TOmada de Decisao Interativa Multicriterio) method. The hybrid geometric TODIM method finds that experiment number 3 is the best and the parameters are as follows: peak current of 2 A, pulse duration of 261 µs, dielectric level of 60 mm, and flushing pressure of 0.7 kg/cm². Sensitivity analysis confirms the robustness of these results, and comparative analysis with existing methods demonstrates the effectiveness of the proposed method in establishing optimal parameters.

**Supplementary Information:**

The online version contains supplementary material available at 10.1038/s41598-025-92713-2.

## Introduction

During the process of machining, a specific type of waste arises, which necessitates careful disposal to protect the environment. In the case of electrical discharge machining, the generated waste could potentially pose a serious health risk to both the operators and the surrounding environment. The gaseous fumes, when inhaled, may cause skin and respiratory problems, thereby leading to detrimental health issues. Stricter regulations from the government have encouraged manufacturers to promote sustainability in their processes to mitigate the adverse effects on health and the environment^1–3^. Adopting optimal process parameters during the machining process can ensure sustainability by minimizing waste generation. When it comes to green die sinking electrical discharge machining, we must consider crucial parameters such as energy consumption and material removal rate to maintain sustainability and prevent any negative environmental effects. We should optimize the process parameters to maintain the objectives of overall machining efficiency. Only a systematic and scientific process can address this challenging decision-making problem.

Multi-criteria decision-making (MCMD) tools can meet the trade-offs and challenges while achieving the overall objectives of the process. There has been a wide array of research studies regarding the application of MCDM tools to optimize the process parameters in the context of electrical discharge machining. The ensuing section provides a comprehensive overview of the research studies that have used different MCDM tools to enhance the machining output of EDM processes.

### Literature survey

The following section provides a comprehensive literature review providing an extensive summary of recent studies on optimizing electrical discharge machining (EDM) parameters to enhance performance across different materials and applications. Vijayakumar and Chandradass^4^ used an integrated CRITIC (*“CRiteria Importance Through Intercriteria Correlation”*)-TOPSIS approach to find the best output process parameters for the wire EDM process, including the rate of material removal, the roughness of the surface, and the profile error, for making gear out of 20MnCr5 steel. They achieved this by considering the following input process parameters: wire feed rate, wire tension, arc off and on time, and servo voltage. We considered each of these input parameters at three levels, and designed 27 Taguchi-based experiments to collect the necessary data. Out of the considered input process parameters, arc on time and wire tension were identified to be the most influential parameters effecting the outcome. In a different study^5^, three MCDM methods—CODAS (*“Combinative Distance-Based Assessment”*), CoCoSo (*“Combined Compromise Solution”*), and MAIRCA (*“Multi-Attribute Ideal-Real Comparative Analysis”*)—were combined with intuitionistic fuzzy (IF) to find the best parameters for the WEDM process in order to balance the cutting speed, surface roughness, and kerf width. We experimented with different levels of pulse-on time, pulse-off time, wire feed, and wire tension. The application of the above approaches revealed the following optimal values of the input process parameters: pulse-on time of 115 µs, pulse-off. A comparative assessment of the considered approaches reveals the robustness of the IF-CoCoSo approach over the others. Tolcha and Lemu^6^ carried out process parameter optimization for EDM of aluminum alloy reinforced with vanadium carbide (VC), a composite. Tolcha and Lemu^6^ considered peak current, discharge voltage, and pulse on-time as the input process parameters and used them to optimize material removal rate (MRR), electrode wear rate (EWR), and surface roughness (SR) using four multi-criteria decision-making (MCDM) methods: Teaching-Learning-Based Optimization (TLBO), VIKOR, Grey Relational Grade (GRG), and the Response Surface Method (RSM). A comparative assessment of the considered approaches revealed the robustness of the TLBO approach over the others. Dhilip et al.^7^ investigated how to make the WEDM process work better for an aluminum matrix composite that was strengthened with boron nitride (BN) and molybdenum disulfide (MoS₂). The researchers used both the entropy weight and VIKOR approaches together to find the best material removal rate (MRR) and surface roughness (SR). The researchers designed the experiments using the L27 orthogonal array, considering the following input parameters: pulse-on time (Ton), pulse-off time (Toff), wire feed (WF), and wire tension (WT). The approach revealed the following optimal values of process parameters: pulse-on time (Ton) of 8 µs, pulse-off time (Toff) of 28 µs, wire feed (WF) of 7 m/min, and wire tension (WT) of 12 g. Karthik et al.,^8^ investigated the composite containing AA6061-T6 and 15wt.% of SiC as matrix and reinforcement respectively for identification of optimal parameters for its EDM machining. To carry out the same, the authors have adopted L27 orthogonal array that considered following input parameters: peak current (IP), pulse on time (Ton) and gap voltage (V). The impact of the said input parameters was analyzed on the following output parameters: material removal rate (MRR), tool wear rate (TWR), circularity (CIR), cylindricity (CYL) and perpendicularity (PP). The optimal process parameters were obtained using the additive ratio assessment (ARAS) approach. The experiment with the following levels of input process parameters was found to be the best: IP = 12 A, Ton = 50 µs and V = 40 V. Optimal process parameters for powder mixed EDM for cylindrical parts fabricated from 90CrSi tool steel was carried out by Phan et al.,^9^. The effect of five input parameters (powder concentration, pulse-on time, pulse-off time, pulse current, and servo voltage) on the Surface Roughness, Electrode Wear Rate and Material Removal Speed was studied using the L18 Taguchi array. Following MCDM methods were employed to assess the optimal process parameters: MABAC (*“Multi-Attributive Border Approximation Area Comparison”*), TOPSIS, Evaluation by an Area-based Method of Ranking (EAMR) methods. Following combination of the process parameters was revealed to yield optimal output response: powder concentration = 0.5 g/L, pulse-on time = 8 µs, pulse-off time = 12 µs, pulse current = 15 A, and servo voltage = 5 V. Chatterjee and Chakraborty^10^ proposed Ranking Alternatives by Median Similarity (RAMS) and RATMI (*“Ranking Alternatives by Trace to Median Index”*) approaches under intuitionistic fuzzy environments to identify the optimal process parameters for EDM processing of Z31 magnesium alloy and Ti-6Al-4 V work material. Grey relational approach was employed by Ranganathan et al.,^11^ to identify the best set of process parameters for EDM of SLMed AlSi10Mg. Following values of the process parameters yielded optimum results: pulse On time = 118 µs, a pulse Off time = 44 µs, servo voltage = 60 V, and a wire feed rate = 7 m s − 1. Chaterjee et al.,^12^ adopted double DNMA (*“normalization-based multiple aggregation”*) approach to identify the appropriate dielectric fluid to carry out deep-hole drilling effectively on aluminum bronze alloy. The most suitable EDM oil was revealed to be Spark SPO amongst the other considered dielectric fluids. The key studies have been examined and summarized as additional findings in Table A1 for quick reference.

### Summary of the literature

- Studies investigate EDM on various materials, including aluminum, titanium, steel alloys, composites, and glass, with electrodes ranging from copper and brass to nickel and titanium-coated tools. These combinations reflect the wide adaptability of EDM for different materials and applications.
- Key input variables across studies include current, pulse duration, feed rate, voltage, wire tension, and other machining parameters. We meticulously control and analyze these factors to improve output measures like material removal rate (MRR), surface roughness, tool wear, and dimensional accuracy.
- The studies apply various optimization and decision-making algorithms, such as Grey Relational Analysis (GRA), TOPSIS, CRITIC, Taguchi methods, and hybrid approaches like MABAC etc., demonstrating a trend toward combining traditional techniques with more complex, multi-objective methods for improved performance and accuracy.
- Common performance metrics include MRR, tool wear rate (TWR), surface quality, recast layer thickness, and heat-affected zone. Methods such as Response Surface Methodology (RSM) and Entropy Weight Methods (EWM) systematically optimize these outputs across studies to assess EDM efficiency.
- The research spans diverse applications from high-precision industrial components to sustainable material processing, showcasing EDM’s relevance across industries, especially in aerospace, automotive, and biomedical sectors where precision and surface integrity are essential.

### Research gaps

The existing literature on EDM reveals several research gaps, particularly in advancing sustainable or green EDM for aluminum grade 5.

- While researchers have extensively studied EDM on a variety of materials, including titanium, steel alloys, composites, and glass, there is a notable gap in the application of green EDM to aluminum grade 5. We have yet to comprehensively explore this material’s unique properties and potential for sustainable manufacturing, particularly in minimizing the environmental impact associated with the EDM process.
- Despite the prevalent use of MCDM techniques like GRA, TOPSIS, and hybrid models (MEREC, MARCOS), few studies have incorporated TODIM—a method that captures the decision-makers’ risk attitudes and trade-offs between conflicting objectives. Using TODIM in EDM for green manufacturing could give more detailed information about how to optimize parameters by considering psychological factors and how important each criterion is different, which would help people make better, more balanced decisions. Most studies currently rely on intuitionistic fuzzy sets to handle linguistic evaluations in EDM parameter optimization. However, exponential-logarithmic single-valued neutrosophic sets (ELSVNS) are a better way to go because they deal with doubt, contradiction, and the complexity that comes with expert judgments better. Integrating ELSVNS could lead to more accurate modeling of expert assessments, thus enhancing the robustness of decision-making frameworks in green EDM.
- While common performance metrics like material removal rate, tool wear rate, and surface quality are well-explored, studies lack a strong focus on environmental impact metrics in EDM for sustainable applications. Green EDM practices could significantly advance with the definition and optimization of specific environmental parameters, particularly in sectors such as aerospace and biomedical engineering that prioritize sustainability.

### The research questions addressed by the presented research work

- RQ1. What are the optimal process parameters (pulse duration, discharge current, dielectric type) for achieving high machining efficiency while minimizing environmental impact in EDM of aluminum grade 5?
- RQ2. How does the use of exponential-logarithmic single-valued neutrosophic sets (ELSVNS) along with the TODIM approach improve the reliability of expert-based EDM parameter optimization??
- RQ3. What specific environmental impact metrics (e.g., carbon footprint, energy efficiency, dielectric fluid toxicity) should be prioritized in green EDM applications?

### The contributions and novelty of the presented research work

- This study addresses the gap in sustainable EDM practices specifically for aluminum grade 5, emphasizing reduced environmental impact and aligning with green manufacturing standards.
- This research uniquely applies the TODIM approach to EDM parameter optimization, considering decision-makers’ risk preferences and criteria trade-offs. This application provides a more comprehensive decision-making framework by reflecting the psychological perspectives of experts involved in green EDM.
- To improve accuracy in linguistic evaluations based on expert judgments, the study incorporates ELSVNS in EDM optimization, addressing the limitations of intuitionistic fuzzy sets. This novel approach enables more effective modeling of indeterminate and contradictory information, which enhances decision-making in green EDM.
- Beyond traditional performance measures, this study incorporates specific environmental metrics in EDM optimization, providing a holistic framework for sustainable practices. This inclusion promotes an eco-friendlier EDM process, which is critical for applications in industries focused on sustainability, such as aerospace and biomedical sectors.

## Preliminaries

This section of the work summarizes the various definitions required for building up the appraisal framework. The single valued neutrosophic set (SVNS), the exponential-logarithmic single valued neutrosophic set, and their score function is talked about in “Preliminaries associated with Neutrosophic sets and its different variants” Section. “Preliminaries associated with aggregation operators” Section summarizes the definitions associated with the hybrid average and geometric operators. “Preliminaries associated with TODIM approach” Section has discussed the definitions associated with the TODIM approach.

### Preliminaries associated with neutrosophic sets and its different variants

#### Definition 2.1

A neutrosophic set (NS) is characterized as follows^39^:

$$\:N=\left\{\left(x,\rho\:\left(x\right), \c{X}\left(x\right),\nu\:\left(x\right)\right)\left|x \epsilon X\right.\right\}$$

ρ(x) indicates the membership degree, X(x) signifies the indeterminacy degree, and ν(x) represents the non-membership degree. Each of these elements takes values in the range ]-0,1+[.

#### Definition 2.2

Equation (1) illustrates the representation of a single-valued neutrosophic set (SVNS)^40,41^:

1 $$\:N=\left\{\left(x,\rho\:\left(x\right), \c{X}\left(x\right),\nu\:\left(x\right)\right)\left|x \epsilon X\right.\right\}$$

where ρ(x), $\c{X}\left(x\right)$, and ν(x) are within the interval [0,1] and satisfy the arithmetic condition: $\:0\le\:\rho\:\left(x\right)+\c{X}\left(x\right)+\:\nu\:\left(x\right)\:\le\:3$.

#### Definition 2.3

The score function for a SVNN can be expressed using Eq. (2) as follows^42^:

2 $$\:S\left(N\right)=\rho\:-\c{X}-\nu\:$$

#### Definition 2.4

An exponential-logarithmic single valued neutrosophic set (ε*Log-*SVNS)^43^ can be expressed using Eq. (3):

3 $$\:SVNN\:\left({\upepsilon\:}L\right)=\left\{\left(x,{\left({e}^{-(-log\rho\:\left(x\right)}\right)}^{\lambda\:},1-{\left({e}^{-(-\text{l}\text{o}\text{g}\left(1-\c{X}\left(x\right)\right)}\right)}^{\lambda\:},1-{\left({e}^{-(-\text{l}\text{o}\text{g}\left(1-\nu\:\left(x\right)\right)}\right)}^{\lambda\:}\right)\left|x \epsilon X\right.,0<\lambda\:\le\:1\right\}$$

In the above expression, the membership function $\:{\left({e}^{-(-log\rho\:\left(x\right)}\right)}^{\lambda\:}\epsilon \left[\text{0,1}\right]$, the indeterminacy degree $\:1-{\left({e}^{-(-\text{l}\text{o}\text{g}\left(1-\c{X}\left(x\right)\right)}\right)}^{\lambda\:}\epsilon \left[\text{0,1}\right]$ and non-membership degree $\:1-{\left({e}^{-(-\text{l}\text{o}\text{g}\left(1-\nu\:\left(x\right)\right)}\right)}^{\lambda\:}\epsilon \left[\text{0,1}\right]$.

### Preliminaries associated with aggregation operators

#### Definition 2.5

Let the SVNNs be denoted by $\:{P}_{i}$. To aggregate the different SVNNs, the exponential logarithmic single valued neutrosophic hybrid average operator can be used and the same can be expressed as^43^:

$$\:\epsilon\:L-SVNHAO\left({P}_{1},{P}_{2},\dots\:{P}_{i}\right)={\oplus\:}_{i=1}^{n}{w}_{i}{P}_{\sigma\:\left(i\right)}$$

$$\:\epsilon\:L-SVNHAO\left({P}_{1},{P}_{2},\dots\:{P}_{i}\right)=\left[1-\prod\:_{i=1}^{n}{\left(1-{W}_{\sigma\:\left(i\right)}\right)}^{{k}_{i}},\prod\:_{i=1}^{n}{\left(1-{H}_{\sigma\:\left(i\right)}\right)}^{{k}_{i}},\prod\:_{i=1}^{n}{\left(1-{L}_{\sigma\:\left(i\right)}\right)}^{{k}_{i}},\right]$$

where $$\:{w}_{i}>0,\sum\:_{i=1}^{n}{w}_{i}=1$$ and $$\:{W}_{\sigma\:\left(i\right)}={\left({e}^{-(-log\rho\:\left(x\right)}\right)}^{\lambda\:}$$, $$\:{H}_{\sigma\:\left(i\right)}={\left({e}^{-(-\text{l}\text{o}\text{g}\left(1-\c{X}\left(x\right)\right)}\right)}^{\lambda\:}$$, $$\:{L}_{\sigma\:\left(i\right)}={\left({e}^{-(-\text{l}\text{o}\text{g}\left(1-\nu\:\left(x\right)\right)}\right)}^{\lambda\:}$$.

#### Definition 2.6

Let the SVNNs be denoted by $\:{P}_{i}$. To aggregate the different SVNNs, the exponential logarithmic single valued neutrosophic hybrid geometric operator can be used and the same can be expressed as^43^:

$$\:\epsilon\:L-SVNHGO\left({P}_{1},{P}_{2},\dots\:{P}_{i}\right)={\otimes\:}_{i=1}^{n}{{P}_{\sigma\:\left(i\right)}}^{{w}_{i}}$$

$$\:\epsilon\:L-SVNHGO\left({P}_{1},{P}_{2},\dots\:{P}_{i}\right)=\left[\prod\:_{i=1}^{n}{\left({W}_{\sigma\:\left(i\right)}\right)}^{{k}_{i}},1-\prod\:_{i=1}^{n}{\left({H}_{\sigma\:\left(i\right)}\right)}^{{k}_{i}},1-\prod\:_{i=1}^{n}{\left({L}_{\sigma\:\left(i\right)}\right)}^{{k}_{i}}\right]$$

### Preliminaries associated with TODIM approach

The essential concept of the TODIM technique is the usage of the prospect value function to measure the dominance or degree of dominance of each alternative relative to the others. In other words, this method evaluates the possibilities by assessing each alternative’s partial and overall dominance over the others. The steps involved in the TODIM method’s calculations have been outlined below^44,45^:

Step 1: In the first phase, the decision matrix U is constructed, where *n* represnets the number of alternatives and *m* the number of criteria.

$$\:\text{U}=\:{\left[{\text{U}}_{\text{i}\text{c}}\right]}_{\text{n}\times\:\text{m}}=\left[\begin{array}{ccc}{x}_{11}&\:\cdots\:&\:{x}_{1m}\\\:\vdots &\:\ddots\:&\:\vdots\\\:{x}_{n1}&\:\cdots\:&\:{x}_{nm}\end{array}\right]$$

(i = 1, 2… n; c = 1, 2… m)

Step 2: The matrix U is normalized using using Eq. (4) for beneficial criteria and using Eq. (5) for the non-beneficial criteria

4 $$\:\text{V}\text{i}\text{j}=\frac{{x}_{ij}}{\sum\:_{i=1}^{n}{x}_{ij}}$$

5 $$\:\text{V}\text{i}\text{j}=\frac{\raisebox{1ex}{$1$}\!\left/\:\!\raisebox{-1ex}{${u}_{ij}$}\right.}{\sum\:_{i=1}^{n}\raisebox{1ex}{$1$}\!\left/\:\!\raisebox{-1ex}{${u}_{ij}$}\right.}$$

where V_ij_= normalized value of x_ij_.

Step 3: The relative weight of the considered criterion fare computed using the reference criteria and the same may be derived in this phase using Eq. (6):

6 $$\:{\text{r}\text{w}}_{\text{c}\text{r}}\:=\frac{{w}_{c}}{{w}_{r}}$$

here *rw*_*cr*_ = relative criteria weight, *w*_*r*_= weight of reference criteria and *w*_*c*_= weight of the criteria.

Step 4: By employing Eq. (7), the dominance level of one alternative (E_i_) over the other alternative (E_j_) is determined.

7 $$\:\delta\:({\text{E}}_{\text{i}},\:{\text{E}}_{\text{j}})\:=\sum\:_{c=1}^{m}{\varPhi\:}_{c\:\:}\left({\text{E}}_{\text{i}},\:{\text{E}}_{\text{j}}\right)$$

where

$$\:{\Phi\:}\text{c}\:({\text{E}}_{\text{i}},\:{\text{E}}_{\text{j}})\:=\left\{\begin{array}{*{20}{c}}\sqrt{\frac{{rw}_{cr}\:({V}_{ic}-{V}_{jc})}{\sum\:_{c=1}^{m}{rw}_{cr}}}\:\:\:\:\:\:\:\:\:\:\:\:\:\:\:\:\:\:\:\:\:if\:\:({V}_{ic}-{V}_{jc})>0\\\:0\:\:\:\:\:\:\:\:\:\:\:\:\:\:\:\:\:\:\:\:\:\:\:\:\:\:\:\:\:\:\:\:\:\:\:\:\:\:\:\:\:\:\:if\:\:\left({V}_{ic}-{V}_{jc}\right)=0\\\:\frac{-1}{\theta\:}\sqrt{\frac{\left(\sum\:_{c=1}^{m}{rw}_{cr}\:\right)({V}_{ic}-{V}_{jc})}{{rw}_{cr}}}\:\:\:\:if\:\:\left({V}_{ic}-{V}_{jc}\right)<0\end{array}\right.$$

where Φ_c_ (E_i_, E_j_) dominance value of alternative E_i_ over E_j_, rw_cr_ = relative criteria weights, and $\:\theta\:$ is the attenuation factor.

The gain and losses of the i^th^ alternative over the j^th^ alternative are represented by $\:({V}_{ic}-{V}_{jc})>0$ and $\:\left({V}_{ic}-{V}_{jc}\right)<0$, respectively.

Step 5: Eq. (8) calculates the degree of total dominance of option E_i_ (£_i_).

8 $$\:{\text{\pounds\:}}_{\text{i}}\:=\frac{\delta\left({E}_{i,\:}{E}_{j}\right)-\text{min}\sum\:_{j=1}^{n}\delta\left({E}_{i,\:}{E}_{j}\right)}{\text{max}\sum\:_{j=1}^{n}\delta\left({E}_{i,\:}{E}_{j}\right)-\text{min}\sum\:_{j=1}^{n}\delta\left({E}_{i,\:}{E}_{j}\right)}$$

Step 6: Considered alternatives are ranked in accordance to the overall dominance values where the alternative having the highest dominance score is deemed as best option.

### Suggested approach

**Fig. 1:**
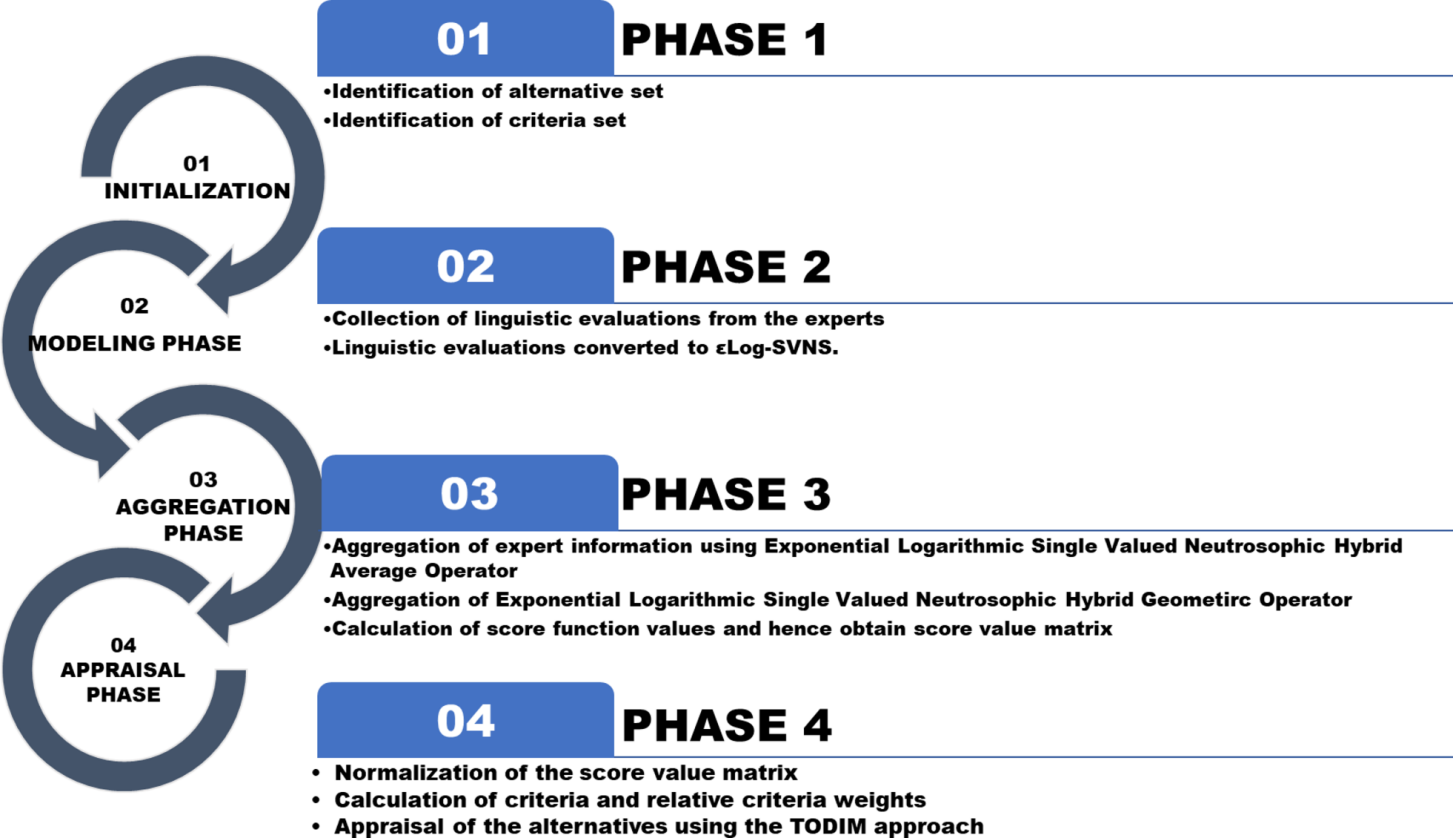
The procedural steps involved in the proposed decision-making framework.

Figure 1 demonstrates the different phases of the proposed decision-making framework which comprises of four phases. These have been discussed as follows:

#### Phase 1: initialization phase

The focus of the proposed decision-making framework shifts to identifying the appropriate criteria and viable alternatives for evaluation to achieve an optimal decision in this phase. The process begins by thoroughly understanding the problem or objective, which informs the selection of relevant criteria. The criteria, which are performance metrics, play a crucial role in evaluating the scrutinized alternatives and should be in line with the case study. Moreover, the identified criteria should be measurable either quantitatively or qualitatively. In the context of the manufacturing process, these criteria can include cost-effectiveness, environmental considerations, resource optimization, and process efficiency in terms of product quality.

In addition to accurate identification of the criteria, identification of appropriate alternatives is equally important. The process can meet its requirements by opting for available options or strategies. One should carefully identify the alternatives, considering the scope of the process or project. Furthermore, one should evaluate and practically implement the identified alternatives. Some examples of alternatives could include policies, waste disposal locations, materials, or experimental strategies.

#### Phase 2: modelling of linguistic evaluations

This phase involves gathering appropriate data from various stakeholders, including shop floor managers, engineers, designers, and architects, among others. The selection of stakeholders is contingent upon the specific project and process under consideration. For example, in the manufacturing process, experts such as machine operators, engineers, and managers can serve as stakeholders, offering valuable technical, environmental, and managerial insights into the ongoing experiments. The stakeholders provide the evaluations for the considered performance metrics either in quantitative or qualitative terms.

After collecting the evaluations, it becomes crucial to model the collected information. In the current context, the Log-SVNS model models the evaluations by transforming them into single-valued neutrosophic values. The process distinguishes itself from others by incorporating an attitude parameter, which enables experts to adjust their evaluations according to their own viewpoints without compromising the accuracy of the results. The parameter provides a level of flexibility, enabling the experts to fully concentrate on expressing their preferences from their perspective, thereby enhancing the benefits of the decision-making process.

In the case of electrical discharge machining, wherein the selection of dielectric fluid plays a major role in the efficiency of the process, the experts may consider important aspects such as cost, wear rate of the tool, material removal rate, and power consumption during the process. Linguistic evaluations, such as “very very high” for the material removal rate, “very very low” for the cost and power consumption, and “very low” for the tool wear rate, can effectively describe the considered aspects. The Log-SVNS model then converts these linguistic terms to neutrosophic values, allowing for flexibility and ensuring that the selected dielectric fluid meets the expectations of experts and all stakeholders.

#### Phase 3: aggregation of the information from the experts

Next, the collected evaluations from the different experts are aggregated next using suitable aggregation operators. In the current scenario, this is achieved by utilizing two operators: (i) the Exponential Logarithmic Single Valued Neutrosophic Hybrid Average Operator and (ii) the Exponential Logarithmic Single Valued Neutrosophic Hybrid Geometric Operator. The primary reason for considering the aforementioned operators is their inclusion of an attitude parameter, a feature absent in other operators. The attitude parameter reflects the preferences of the experts and hence lends flexibility and adaptability in the decision-making process. Moreover, the inclusion of an attitude parameter is critical to precisely capture experts’ perspectives regarding the considered criteria and hence aggregate the opinions that resonate with the opinions of all the experts involved in the decision-making process.

In addition to the inclusion of an attitude parameter, the operators have the potential to capture the non-linear relationship between the different performance measures. The said operators are also useful because they combine exponential and logarithmic functions, which makes it possible to model dynamic features like growth, consistency, and decay that happen in real-life decision-making.

On completion of the aggregation process, the outcome is a unified decision matrix that contains evaluations only in terms of neutrosophic numbers. This matrix serves as a precursor to further calculations for the score functions.

#### Phase 4: ranking phase

The ranking phase follows the aggregation phase, evaluating the identified and scrutinized alternatives through a rigorous process. The normalization process, which reduces the score value decision matrix to a scale between 0 and 1, initiates this phase. This is one of the crucial steps in the ranking phase since it enables the comparison of the data in the score value matrix after removing the scales, i.e., the different measurement units possessed by the performance measures. This leads to fair and precise evaluations of the considered alternatives and hence clarity on the outcome of the decision-making process.

In the second step of the ranking phase, the normalized decision matrix is employed to obtain the weights and the relative criteria weights. Experts can either subjectively calculate these criteria weights based on their perspective or objectively, using methods like entropy. The derived values of the criteria weights are used to calculate the relative criteria weights, comparing all the criteria weights to the reference weight, which is the maximum of all the weights. The weight values are then used to calculate the prospect value scores. The prospect value score integrates both the positive and negative evaluations, providing a comprehensive assessment of each alternative’s overall potential. The alternatives are ranked based on these prospect value scores. The alternative with the highest prospect value score is considered the most optimal, as it represents the smallest aggregate deviation from the ideal criteria, reflecting the best balance between advantages and drawbacks.

## Application of proposed method

**Fig. 2:**
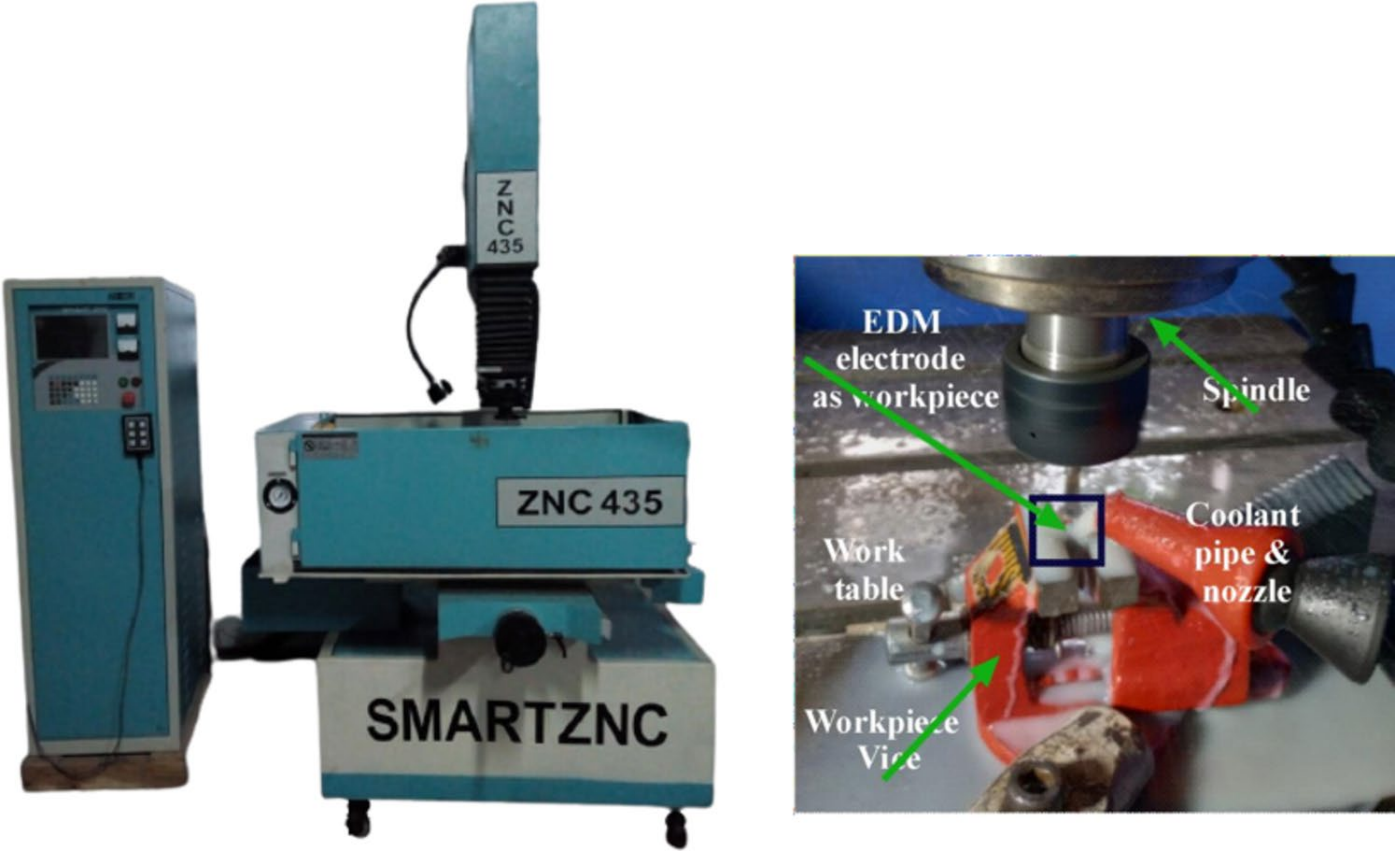
Experimental setup.

This section explains the application of the proposed hybrid MCDM-based approach for green die-sinking EDM. In EDM, an electric arc between two electrodes generates the energy needed for material removal and is conducted in a dielectric medium (foam water) using a copper electrode on aluminum grade 5 (see Fig. 2).

In the initial phase of the proposed decision-making framework, a suitable set of alternatives and evaluation criteria were established. The nine experiments, designed based on the design of experiments (DOE) methodology, were considered as alternatives (Table 1). Table 1 details the input process variables—peak current (A), pulse duration (µs), dielectric level (mm), and flushing pressure (kg/cm²)—and their levels, selected through an extensive literature review. The criteria set includes sustainability-oriented factors: technical criteria such as process time (s) (C1) and relative tool wear ratio (REWR) (C2), and environmental criteria like process energy (W) (C3), dielectric consumption (cm³) (C4), and aerosol concentration (mg/m³) (C5).

The input parameters were selected based on the literature survey and considering their significant impact on the EDM process outputs i.e., machinability performance and environmental sustainability. The intensity of the electric discharge is governed by peak current value. Higher the peak current, higher the intensity of the electric discharge and hence higher the material removal rate. However, the increase in peak current value results in increased electrode wear and energy usage. The pulse duration governs the duration of the electric discharge with longer duration of pulse leading to higher material removal rate but at the same time resulting in thermal damage and higher consumption of energy. Levels of dielectric fluid controls the cooling efficiency, removal of debris and generation of aerosol. Thermal stresses and generation of toxic by products can be kept under check with appropriate dielectric levels. The effective removal of eroded material is ensured by maintaining appropriate flushing pressure. Poor machining may be outcome of inadequate flushing pressure which also results in degradation of dielectric fluid.

**Fig. 3:**
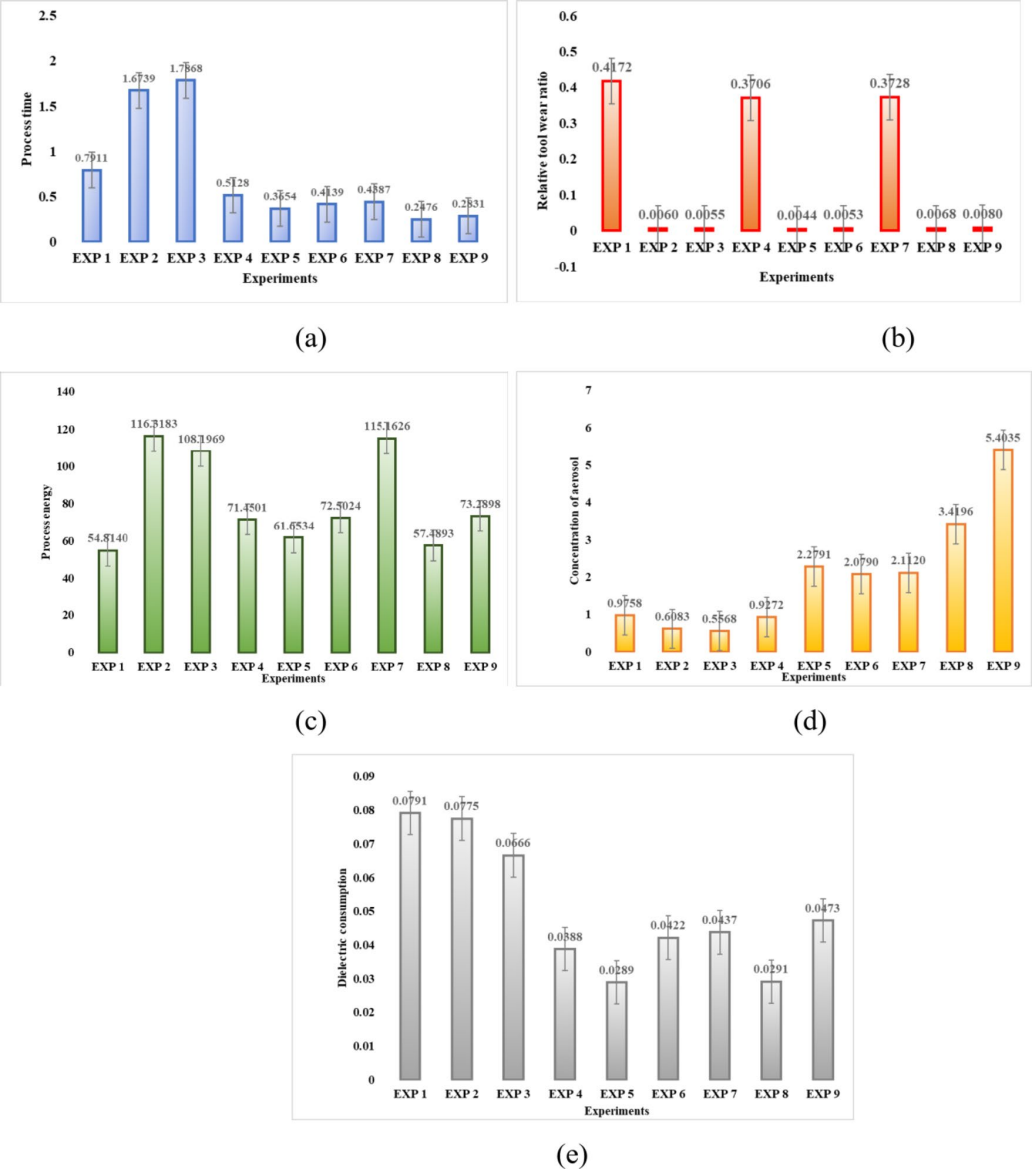
The output process response (**a**) process time(s), (**b**) relative tool wear ratio (REWR), (**c**) process energy (W), (**d**) dielectric consumption (cm^3^) and (**e**) concentration of aerosol (mg/m^3^).

Material removal rate (MRR) affects both process time and operational costs. In EDM operations, electrode wear significantly impacts operational expenses and waste generation. The relative tool wear ratio (REWR) measures the tool wear relative to the workpiece wear. Process energy, which has environmental implications, is determined by the energy consumed during EDM, influenced by the gap voltage, discharge current, and duration of current flow. Exposure to toxic aerosols is a major occupational hazard, particularly when hydrocarbon dielectric fluids are used, as they generate metallic particles and reaction by-products. Figure 3 shows the experimentally obtained output responses for each criterion for the experiments listed in Table 1.

**Table 1:** Design of experiments and the alternatives for the appraisal process^45^.

Exp. No.	Peak current	Pulse duration	Dielectric level	Flushing pressure
1	2	2	40	0.3
2	2	261	60	0.5
3	2	520	80	0.7
4	4.5	2	60	0.7
5	4.5	261	80	0.3
6	4.5	520	40	0.5
7	7	2	80	0.5
8	7	261	40	0.7
9	7	520	60	0.3

In *phase 2* of the proposed approach, experts provide linguistic evaluations of the experimental outcomes based on the established criteria and generated data. Table 2 presents the linguistic evaluations from one of the experts. These evaluations are then converted into single-valued neutrosophic numbers as shown in Table 3. Subsequently, these single-valued neutrosophic numbers are transformed into εLog-SVNNs, which are displayed in Table 4.

**Table 2:**
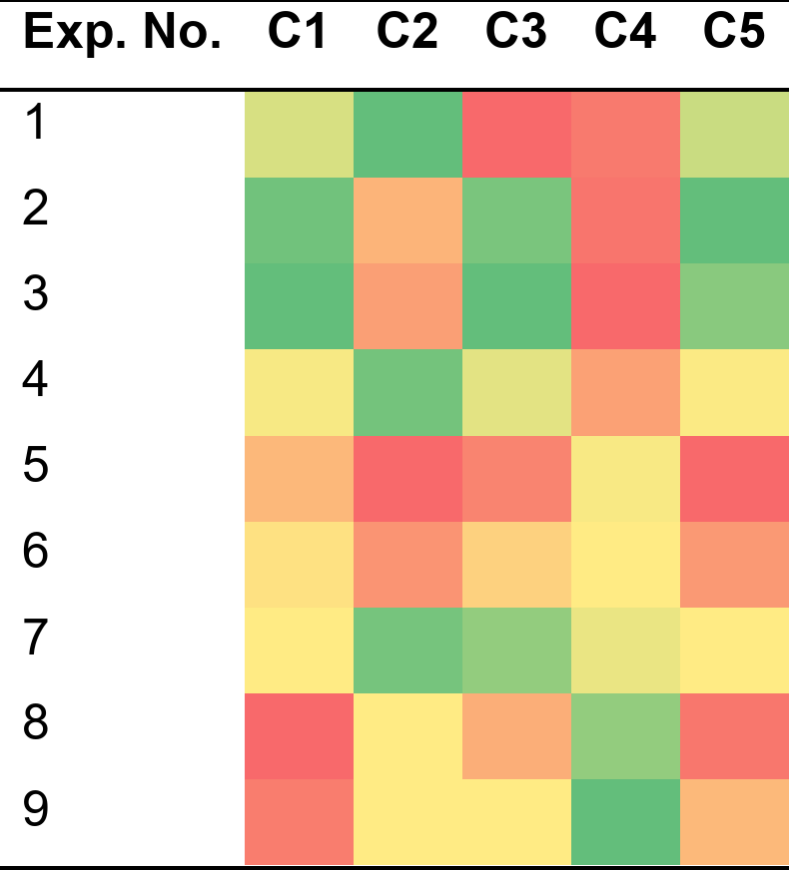
Evaluations from expert 1.

**Table 3:**
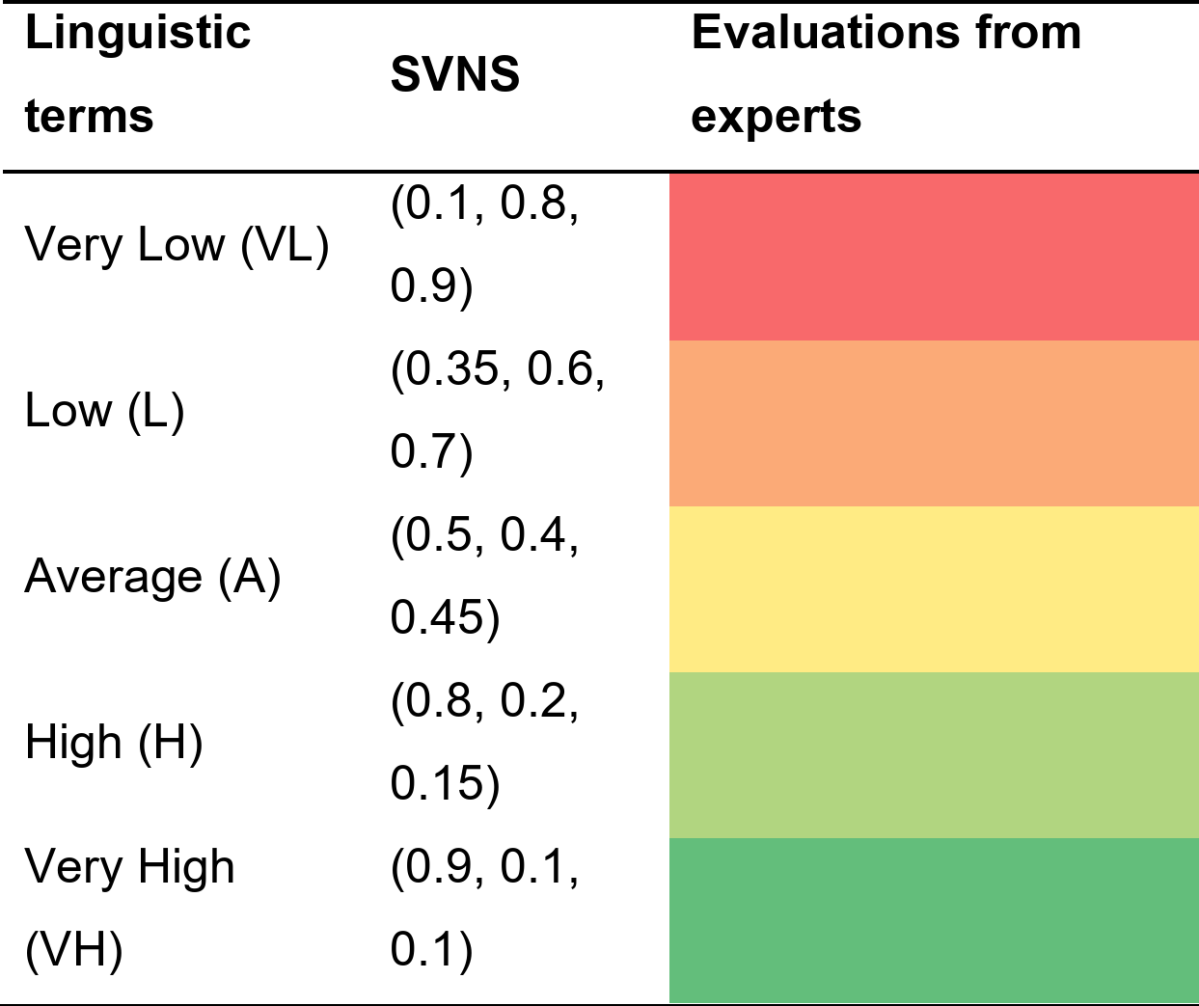
Linguistic scale for SVNS[55].

**Table 4:** Linguistic scale for SVNS.

Exp No.		C1			C2			C3	
1	0.95	0.05	0.03	0.98	0.02	0.02	0.61	0.29	0.39
2	0.98	0.02	0.02	0.80	0.18	0.23	0.98	0.02	0.02
3	0.98	0.02	0.02	0.80	0.18	0.23	0.98	0.02	0.02
4	0.86	0.10	0.12	0.98	0.02	0.02	0.86	0.10	0.12
5	0.80	0.18	0.23	0.61	0.29	0.39	0.61	0.29	0.39
6	0.86	0.10	0.12	0.80	0.18	0.23	0.86	0.10	0.12
7	0.86	0.10	0.12	0.80	0.18	0.23	0.98	0.02	0.02
8	0.61	0.29	0.39	0.98	0.02	0.02	0.80	0.18	0.23
9	0.80	0.18	0.23	0.86	0.10	0.12	0.86	0.10	0.12

In Phase 3 of the proposed decision-making framework, experts’ linguistic evaluations are consolidated using the exponential logarithmic single-valued neutrosophic hybrid average operator and the exponential logarithmic single-valued neutrosophic hybrid geometric operator. Table 5 presents the aggregated matrix derived from the hybrid average operator, while Table 6 shows the matrix obtained with the hybrid geometric operator. Following this, score values are computed and displayed in Tables 7 and 8, corresponding to the exponential logarithmic single-valued neutrosophic hybrid average operator and the hybrid geometric operator, respectively.

**Table 5:** Aggregated matrix obtained using exponential logarithmic single valued neutrosophic hybrid average operator.

Exp No.		C1			C2			C3	
1	1.00	0.86	0.90	1.00	0.93	0.93	0.94	0.35	0.22
2	1.00	0.91	0.92	0.99	0.60	0.52	1.00	0.93	0.93
3	1.00	0.93	0.93	0.99	0.55	0.46	1.00	0.93	0.93
4	1.00	0.72	0.68	1.00	0.93	0.93	1.00	0.72	0.68
5	0.99	0.55	0.46	0.94	0.35	0.22	0.97	0.41	0.28
6	1.00	0.72	0.68	0.99	0.55	0.46	1.00	0.72	0.68
7	1.00	0.72	0.68	1.00	0.78	0.74	1.00	0.89	0.91
8	0.94	0.35	0.22	1.00	0.78	0.75	0.99	0.55	0.46
9	0.98	0.47	0.36	1.00	0.72	0.68	1.00	0.72	0.68

**Table 6:** Aggregated matrix obtained using exponential logarithmic single valued neutrosophic hybrid geometric operator.

Exp No.		C1			C2			C3	
1	0.86	1.00	1.00	0.93	1.00	1.00	0.22	0.97	0.94
2	0.91	1.00	1.00	0.55	1.00	0.99	0.93	1.00	1.00
3	0.93	1.00	1.00	0.50	0.99	0.99	0.93	1.00	1.00
4	0.64	1.00	1.00	0.93	1.00	1.00	0.64	1.00	1.00
5	0.50	0.99	0.99	0.22	0.97	0.94	0.29	0.98	0.96
6	0.64	1.00	1.00	0.50	0.99	0.99	0.64	1.00	1.00
7	0.64	1.00	1.00	0.76	1.00	1.00	0.89	1.00	1.00
8	0.22	0.97	0.94	0.72	1.00	1.00	0.50	0.99	0.99
9	0.38	0.99	0.98	0.64	1.00	1.00	0.64	1.00	1.00

**Table 7:** Score value matrix obtained using exponential logarithmic single valued neutrosophic hybrid average operator.

Exp. No.	C1	C2	C3	C4	C5
1	–1.14	–1.07	–1.69	–1.69	–1.14
2	–1.09	–1.44	–1.07	–1.69	–1.07
3	–1.07	–1.48	–1.07	–1.69	–1.11
4	–1.36	–1.07	–1.36	–1.48	–1.36
5	–1.48	–1.69	–1.66	–1.36	–1.69
6	–1.36	–1.48	–1.36	–1.36	–1.48
7	–1.36	–1.24	–1.11	–1.36	–1.36
8	–1.69	–1.28	–1.48	–1.11	–1.69
9	–1.59	–1.36	–1.36	–1.07	–1.48

**Table 8:** Score value matrix obtained using exponential logarithmic single valued neutrosophic hybrid geometric operator.

Exp. No.	C1	C2	C3	C4	C5
1	–0.76	–0.87	0.37	0.37	–0.76
2	–0.83	–0.13	–0.87	0.37	–0.87
3	–0.87	–0.02	–0.87	0.37	–0.80
4	–0.40	–0.87	–0.40	–0.02	–0.40
5	–0.02	0.37	0.28	–0.40	0.37
6	–0.40	–0.02	–0.40	–0.40	–0.02
7	–0.40	–0.52	–0.80	–0.40	–0.40
8	0.37	–0.54	–0.02	–0.80	0.37
9	0.15	–0.40	–0.40	–0.87	–0.02

Phase 4 then appraises the considered experiments (alternatives), identifying the optimal process parameters. As a first step to this phase, the score value matrix obtained using the exponential logarithmic single valued neutrosophic hybrid average operator and the exponential logarithmic single valued neutrosophic hybrid geometric operator is normalized. The normalized values are then used to obtain the overall prospect theory of each experiment and hence the appraisal results.

## Results and discussions

**Fig. 4:**
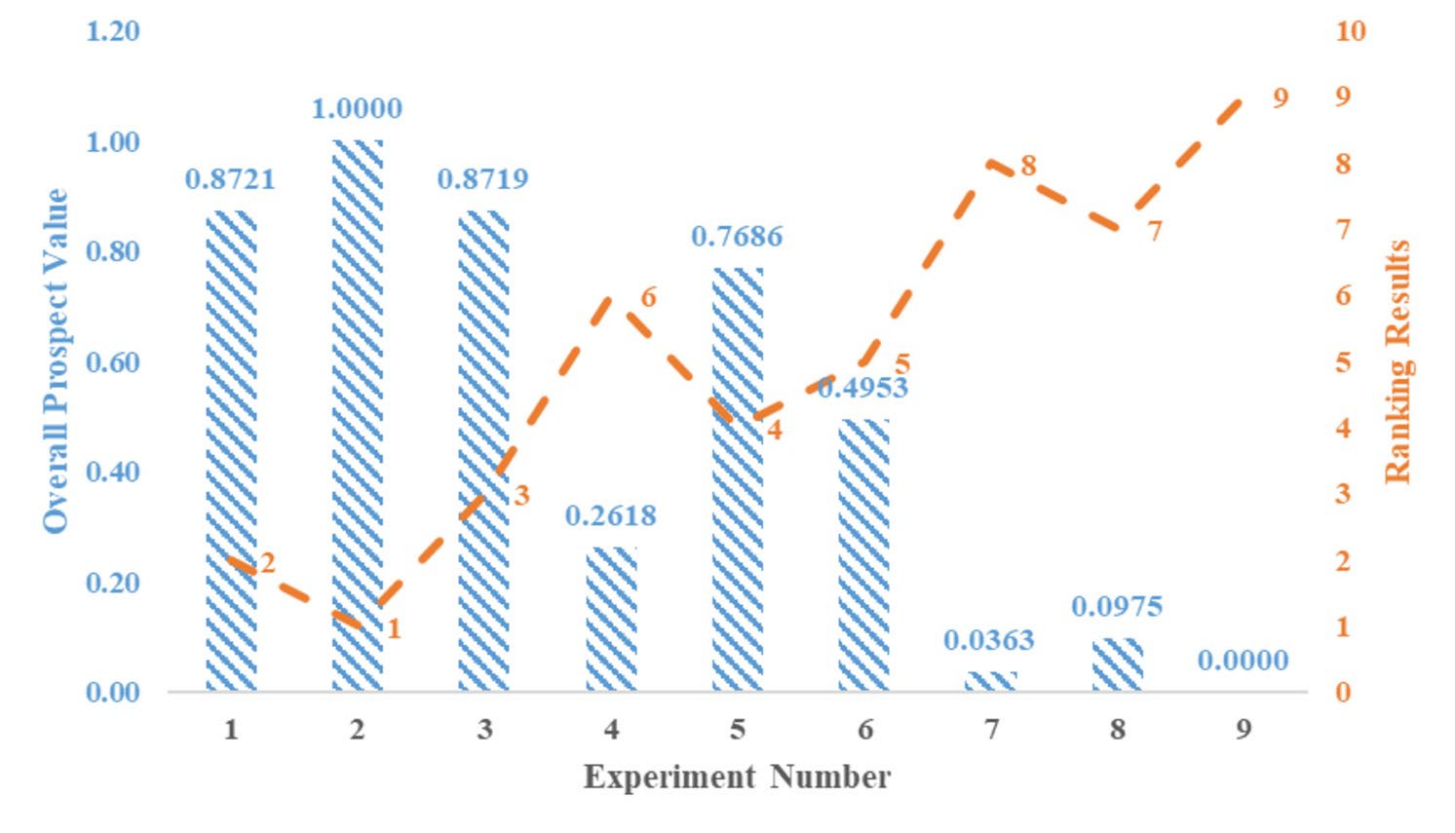
Ranking outcomes using the exponential logarithmic single-valued neutrosophic hybrid geometric TODIM approach.

**Fig. 5:**
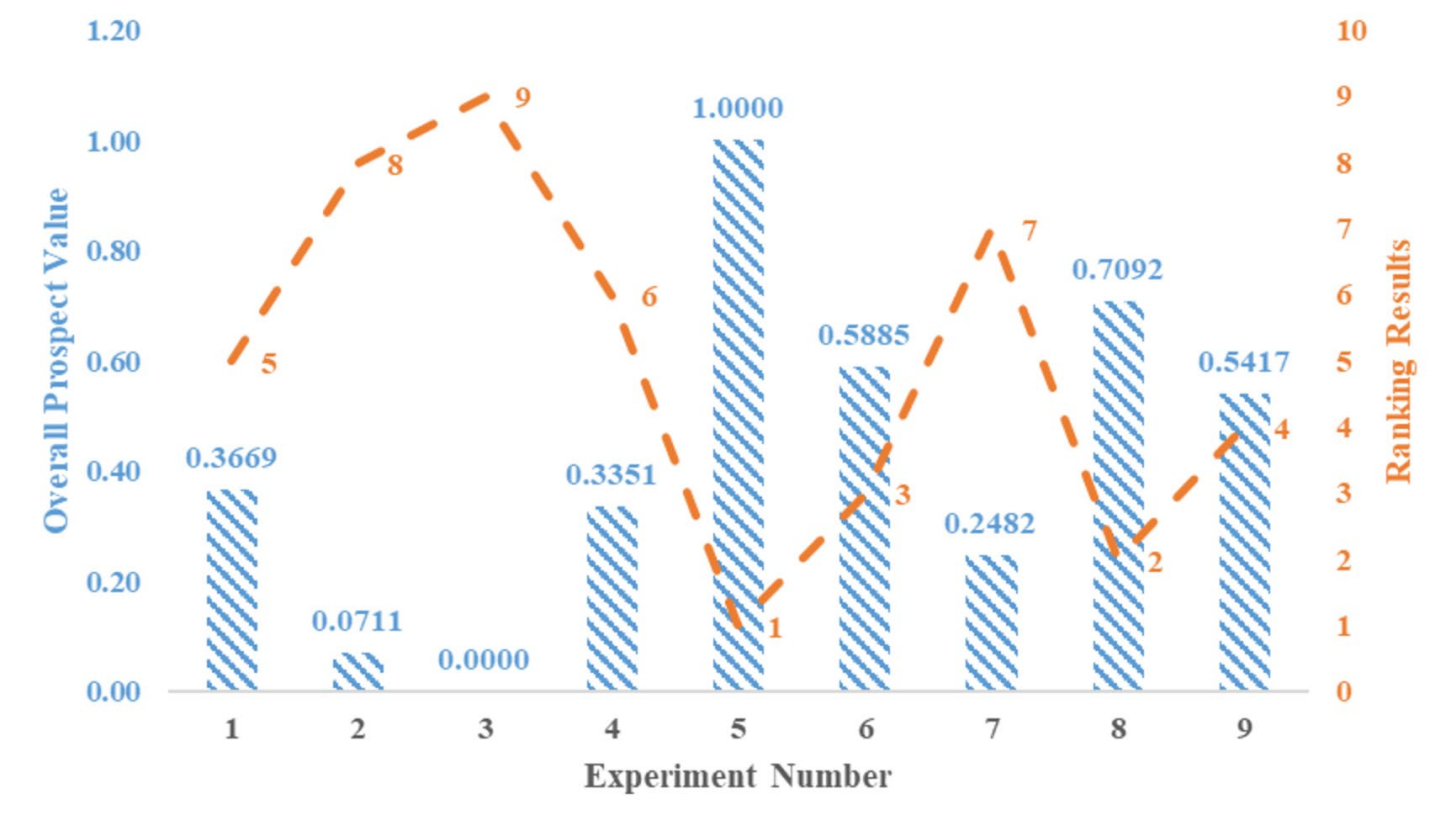
Ranking outcomes using the exponential logarithmic single-valued neutrosophic hybrid averaging TODIM approach.

Figures 4 and 5 respectively display the results obtained using the exponential logarithmic single-valued neutrosophic hybrid average TODIM and hybrid geometric TODIM approaches. The picture (Fig. 4) shows that Experiment 3, with peak current of 2 A, pulse duration of 520 µs, dielectric level 80 mm, and flushing pressure 0.5 kg/cm², has the highest prospect value score of 1, making it the best experiment in the exponential logarithmic single-valued neutrosophic hybrid geometric TODIM method. These experiments are ranked in the following order: Exp 3 > Exp 2 > Exp 7 > Exp 4 > Exp 1 > Exp 9 > Exp 6 > Exp 8 > Exp 5. According to the exponential logarithmic single-valued neutrosophic hybrid average TODIM approach (Fig. 5), Experiment 2, with parameters of peak current 2 A, pulse duration 261 µs, dielectric level 60 mm, and flushing pressure 0.7 kg/cm², has the highest prospect value score of 1, making it the best among the experiments. The ranking order for this approach is: Exp 2 > Exp 1 > Exp 3 > Exp 5 > Exp 6 > Exp 4 > Exp 8 > Exp 7 > Exp 9. Sensitivity analysis regarding criteria weights and the attenuation factor has been conducted to assess the robustness of these ranking results for both approaches. Experiments 4, 5, 6, 7, 8, and 9 show significantly lower process times, suggesting that higher peak currents and optimal flushing conditions improve efficiency. Experiments 2, 3, 5, and 6 show minimal tool wear, with Experiment 5 having the lowest REWR (0.0041). This suggests that moderate peak currents and well-optimized dielectric levels contribute to tool sustainability. Experiments 1, 2, and 3 have the lowest aerosol concentrations, implying that lower peak currents and well-regulated flushing contribute to cleaner machining. Experiment 5 has the lowest dielectric consumption (0.0332 cm³), demonstrating that balancing current and dielectric level optimizes fluid use.

### Sensitivity analysis

#### Sensitivity analysis W.r.t the criteria weights

The robustness and reliability of the ranking results is one of the important aspects of any decision-making framework. This can be addressed through the sensitivity analysis approach, and the same can be carried out using the following ways: The first method involves assessing the sensitivity of the ranking results derived from the criteria weights, while the second method involves analyzing the sensitivity of the obtained rankings using the attenuation factor. Criteria weights represent the importance attached to each criterion while making decisions. Therefore, modifications to these criteria could potentially impact the ranking results. Therefore, we can vary the criterion weights for each considered criterion through a systematic process, and subsequently analyze the impact on the rankings.

**Fig. 6:**
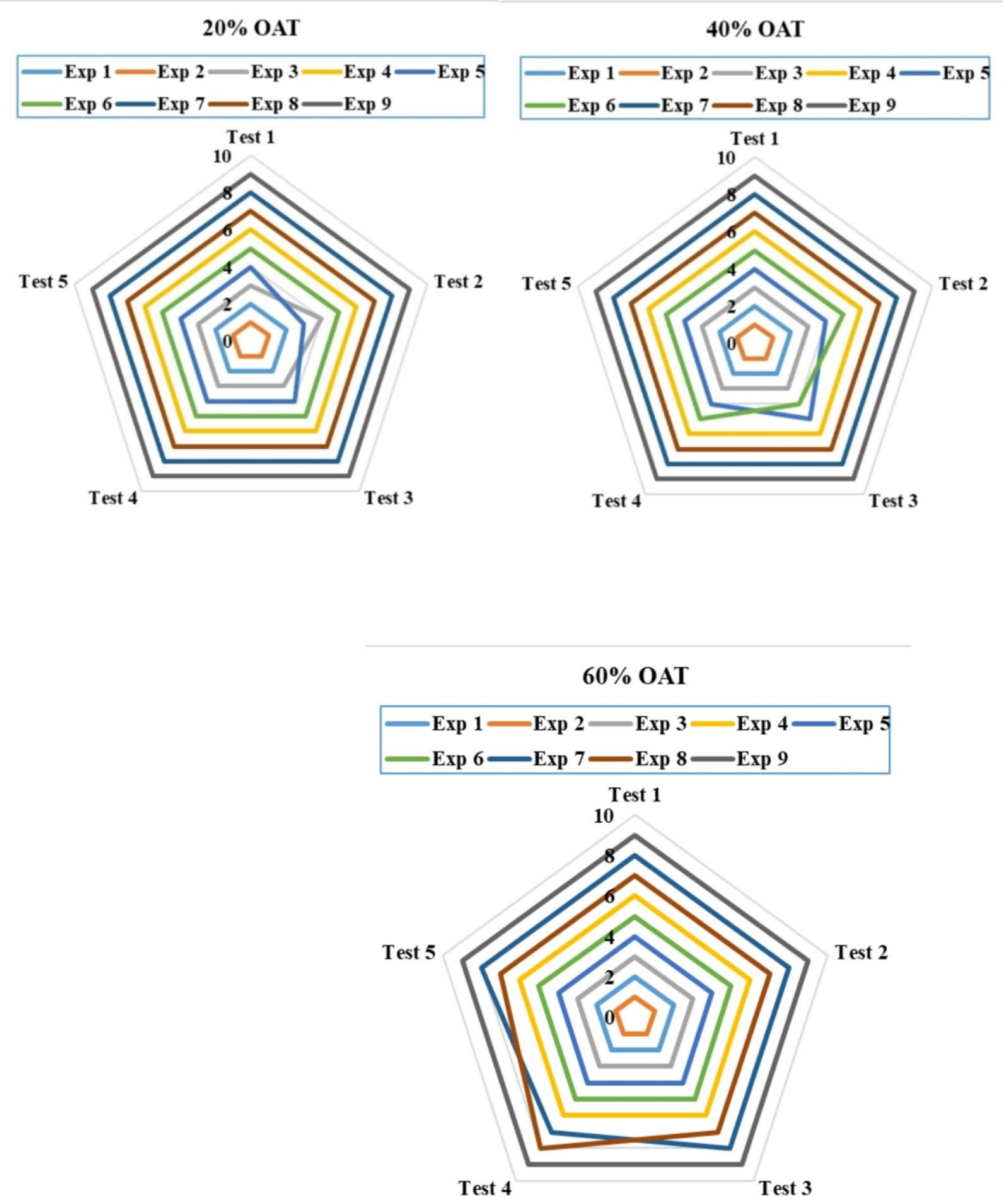
Sensitivity analysis of ranking outcomes based on criteria weights using the exponential logarithmic single-valued neutrosophic hybrid geometric TODIM approach.

**Fig. 7:**
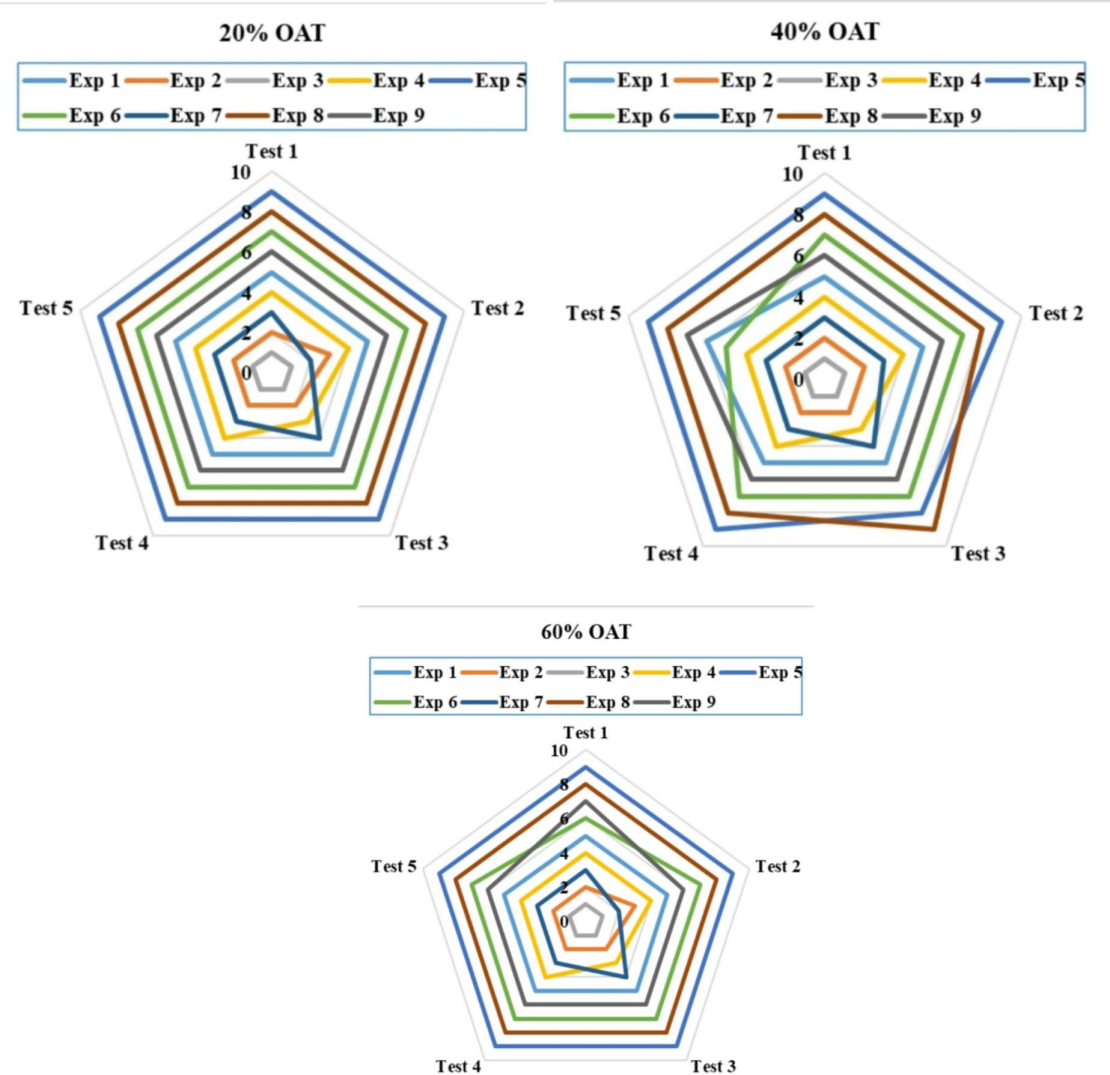
Sensitivity analysis of ranking outcomes relative to criteria weight adjustments using the exponential logarithmic single-valued neutrosophic hybrid average TODIM approach.

The presented work uses a one-at-time (OAT) approach to vary the criterion weights, independently adjusting them by 20%, 40%, and 60% for each considered criterion. This approach allows for a comprehensive understanding of each criterion’s impact, as it allows for the isolation of each criterion individually, eliminating the compounding effect of other criteria, and subsequently assessing its influence on the ranking results. Both suggested methods—the averaging and geometric TODIM methods—were used. The results can be seen in Fig. 6 for the averaging TODIM method and in Fig. 7 for the geometric TODIM method. As evident from the visual representation, the ranking results are robust regarding the variation in criterion weights.

Despite significant changes in the criteria weights, the geometric TODIM approach consistently produces stable ranking results. The outcomes therefore suggest that the geometric TODIM approach yields result that are highly consistent with the criteria for weight variability. The presence of a geometric operator may be one of the reasons behind the stability of the approach, as it has the potential to smooth out the impact of the changing values of the criteria weights. However, the averaging TODIM approach shows noticeable variations in the ranking outcomes. Although the variations are not significant, they clearly indicate a certain level of sensitivity to the criteria weights. Experts may prefer this level of sensitivity when they want the framework to react to even minor modifications in the criteria weights.

Overall, the results of the sensitivity analysis, considering the weights of the criteria, demonstrate the superiority of the geometric TODIM approach over the averaging approach. Moreover, it is the discretion of the experts to opt for a suitable approach that can suit their requirements.

#### Sensitivity analysis W.r.t the Attenuation factor

Sensitivity analysis of the ranking outcomes based on the attenuation factor is another critical aspect of the decision-making process in the context of the risk appetite of the experts. The attenuation factor, which reflects the experts’ psychological behavior towards risk, plays a crucial role in assessing the variation in the ranking results as the attenuation factor changes.

Different values of the attenuation factor signify different levels of the experts’ risk-digesting abilities. When the attenuation factor values fall between 0 and 1, it indicates that the expert is highly averse to taking risks, suggesting that they prioritize potential gains over potential losses. A value of 1 for the attenuation factor signifies that the expert has a neutral stance, i.e., they have the potential to balance risk aversion and tolerance. The value of the attenuation factor greater than 1 show that the expert has the potential to take more risk to gain higher prospective rewards.

In the present study, the values of attenuation factor are considered at the following levels: 0.1, 1, 2, 5, and 10. As previously discussed, the levels of attenuation factor considered will encompass the full range of psychological behaviors exhibited by experts in relation to their risk appetite. The variations in ranking outcomes with the attenuation factor provide an insight into the stability of the outcomes. If the outcomes are stable, then it is suggestive of the robustness of the proposed approach, while higher variations in the ranking outcomes are suggestive of a greater influence of the risk appetite on the decision-making process.

**Fig. 8:**
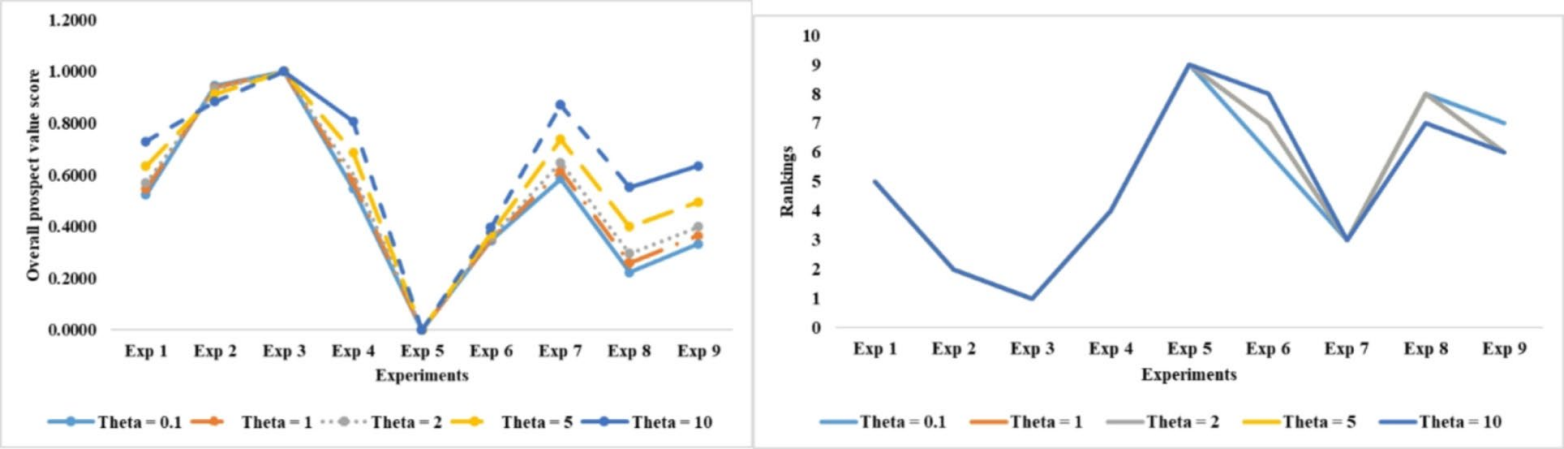
The ranking results outcomes based on different levels of the attenuation factor using the geomtric TODIM approach.

**Fig. 9:**
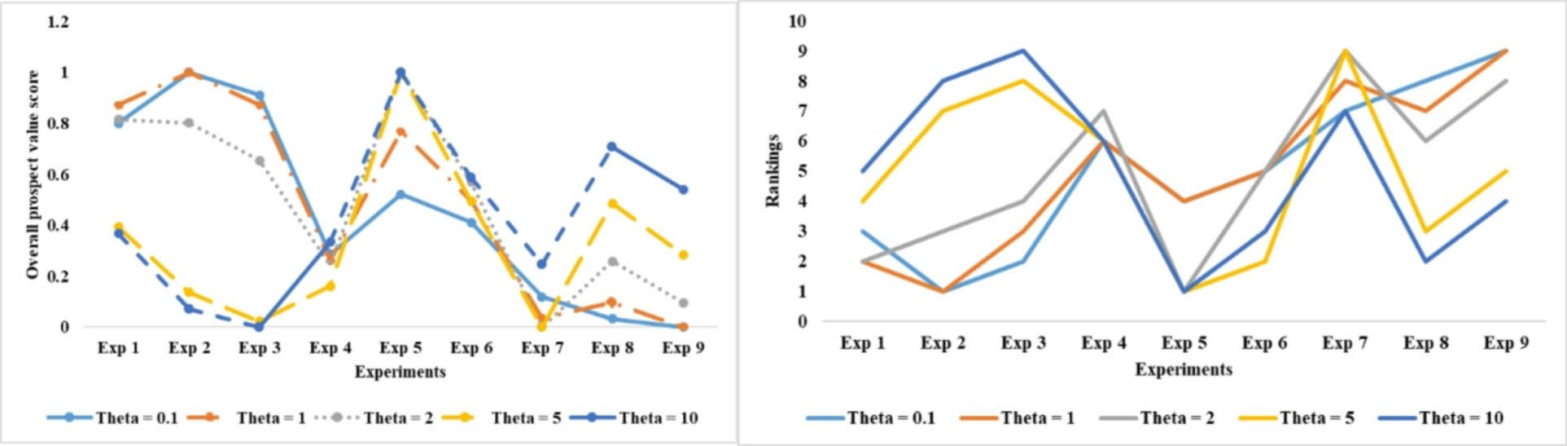
The ranking results outcomes based on different levels of the attenuation factor using the averaging TODIM approach.

Figures 8 and 9 show the ranking outcomes based on different levels of attenuation factor, using the averaging and geometric TODIM approaches. These figures plot the overall prospect values and, consequently, the ranking outcomes as the attenuation factor changes. The depictions clearly show slight shifts in the ranking outcomes for the geometric TODIM approach, indicating the stability of these results. The geometric operator, which smoothes out the extremes and provides a more balanced view, may be responsible for this. On the other hand, the average TODIM approach shows greater sensitivity to the changes in the levels of the attenuation factor.

Differences in the stability of the ranking outcomes for the considered decision-making approaches are indicative of the degree of robustness for both approaches. Experts may choose the geometric TODIM approach due to its higher level of robustness, as they anticipate it will lessen the impact of variable risk appetite. As a result, in cases where the subjective risk appetite can lead to variations, such as in sensitive decisions, the approach may be preferable.

### Comparative analysis

**Fig. 10:**
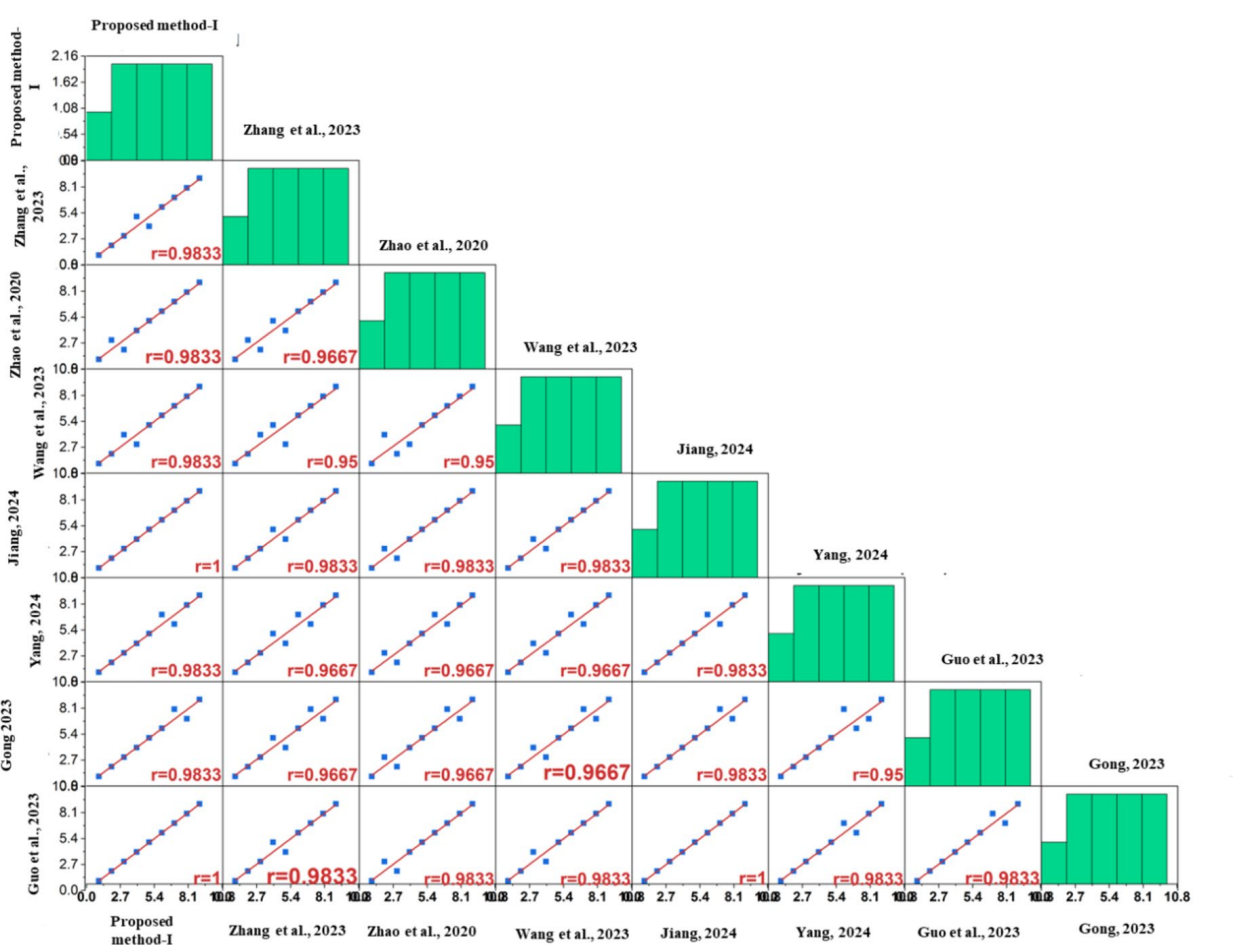
Comparative analysis of the ranking results obtained using the proposed approach and other existing.

The geometric TODIM approach has been compared with existing decision-making frameworks to clearly reveal the advantages and limitations of the proposed approach. To accomplish this, the considered case study has also been solved using the following approaches: The case study was solved using the following approaches: 2-tuple linguistic neutrosophic TODIM^46^, Type-2 neutrosophic fuzzy TODIM^47^, LogTODIM neutrosophic fuzzy TODIM^48^, Exponential neutrosophic fuzzy-TODIM^49^, Triangular Fuzzy Neutrosophic-TODIM^50^, double-valued neutrosophic fuzzy TODIM^51^, and linguistic neutrosophic cloud fuzzy TODIM^52^. Figure 10 depicts the comparative assessment, which indicates a higher degree of consistency. Despite slight variations in the ranking results, experiment 3 continues to be the best among all considered approaches. The slight variations may be attributed to the type of fuzzy set employed to model the linguistic evaluations. The comparative analysis highlights the following salient points:

1. The proposed approach as well as the 2-tuple linguistic neutrosophic fuzzy TODIM approach^46^ have the potential ability to manage uncertainty in the decision-making process, but they tend to serve different contexts. On the one hand, the proposed approach integrates exponential and logarithmic functions to model truth, falsity, and indeterminacy, making it suitable for situations with a high degree of ambiguity. On the other hand, the 2-tuple linguistic neutrosophic fuzzy TODIM approach simplifies intuitive expressions, making it more suitable for cases where linguists dominate. Although the proposed approach is superior to fine-tuning the ambiguities, it is computationally complex in comparison to the 2-tuple linguistic neutrosophic fuzzy TODIM approach. In totality, the proposed approach is advantageous over the 2-tuple linguistic neutrosophic fuzzy TODIM approach in terms of the precision of the outcomes.
2. It is possible for both approaches to model ambiguities accurately when compared to the Type-2 neutrosophic fuzzy TODIM approach^47^. The main difference is the level of sophistication used for modeling. The proposed approach achieves precision by integrating exponential and logarithmic functions to capture falsity, truth, and indeterminacy, thereby enhancing control over uncertainties. However, this comes at the cost of computational complexity. On the other hand, the precision of the Type-2 neutrosophic fuzzy TODIM approach primarily stems from its potential to handle situations with deeper uncertainty, as Type-2 neutrosophic sets are capable of handling a higher degree of ambiguity. However, the said approach is relatively more challenging to be implemented in comparison to the proposed approach. On the one hand, the proposed approach provides flexibility, while the Type-2 neutrosophic fuzzy TODIM approach covers a wider spectrum of uncertainty.
3. Based on its use of only logarithmic functions to model uncertainties, the Logarithmic Single Valued Neutrosophic Fuzzy TODIM approach^48^ is easier on computers than the proposed approach. On the other hand, the proposed approach integrates both exponential and logarithmic functions to model the uncertainties, which is the primary reason for its computational complexity. Despite its computational complexity, the proposed approach accurately models falsity, indeterminacy, and truth, even in complex dynamics situations. The Logarithmic Single Valued Neutrosophic Fuzzy TODIM approach, on the other hand, falls short in terms of precision and flexibility.
4. The Exponential Neutrosophic Fuzzy TODIM approach^49^ is computationally simpler in comparison to the proposed approach since it uses only exponential functions to model the uncertainties. On the other hand, the proposed approach integrates both exponential and logarithmic functions to model the uncertainties, which is the primary reason for its computational complexity. Despite its computational complexity, the proposed approach accurately model’s falsity, indeterminacy, and truth, even in complex dynamics situations. On the other hand, the exponential single-valued neutrosophic fuzzy TODIM approach does not possess this level of precision, but it can be utilized in scenarios where precision is not the primary concern, but rather the computational speed is crucial.
5. When compared to the triangular fuzzy fuzzy TODIM approach^50^, the proposed approach excels because it has the potential to accurately model the linguistic evaluation in situations with deep ambiguities. Although the triangular fuzzy approach is simpler in implementation, this comes at the expense of precisely handling the uncertainty in the process.
6. The Double-Valued Neutrosophic Fuzzy TODIM approach^51^ models the uncertain information through two sets of values. While this may simplify the calculation and the framework, it also limits the ability of the framework to capture intricate uncertainties. On the other hand, the proposed approach effectively handles and controls the model uncertainty by integrating exponential and logarithmic functions. Therefore, the proposed approach is superior to the double-valued approach as it can handle complex and dynamic intricacies more effectively.
7. The Linguistic Neutrosophic Cloud Fuzzy TODIM approach^52^ focuses on capturing uncertainty through linguistic terms and cloud models, providing intuitive, human-understandable assessments that are easier to interpret but less mathematically rigorous. While the linguistic approach excels in qualitative, human-centric decisions, it lacks the computational depth and flexibility of the proposed model, making it less suitable for highly quantitative or detailed analyses.

## Conclusions

This research provides a novel framework for sustainable optimization of Electrical Discharge Machining (EDM) parameters, particularly for aluminum grade 5, addressing a critical gap in green EDM practices. By aligning the EDM process with green manufacturing standards and integrating specific environmental metrics, this study goes beyond conventional performance measures to incorporate sustainability directly into the optimization framework. This holistic approach supports eco-friendly EDM practices and is especially relevant for industries focused on sustainability, such as aerospace and biomedical sectors.

One of the major contributions of the presented work is the application of the TODIM approach to carry out parametric optimization of the green die sinking EDM process for aluminum grade 5 material. Through the TODIM approach, the risk-digesting capability of the experts is considered, which is one of the influential and critical factors in a decision-making process. Furthermore, this approach enhances the robustness of the outcomes, thereby improving the sustainability aspects of EDM. Another important consideration that the proposed framework takes into account is the use of Exponential Logarithmic Single-Valued Neutrosophic Sets (ELSVNS) to model the linguistic evaluations from experts. The inclusion of Exponential Logarithmic Single-Valued Neutrosophic Sets (ELSVNS) overcomes the limitations of the commonly used intuitionistic fuzzy sets, as the integration of exponential and logarithmic functions allows for the precise modeling of conflicting information. Additionally, the exponential logarithmic single-valued neutrosophic hybrid average operator and the exponential logarithmic single-valued neutrosophic hybrid geometric operators have also been integrated to lend flexibility and adaptability into the decision-making process through the inherit attitude parameter in the operators.

The results of the approaches indicate that experiment 3 and experiment 2, which were obtained using the exponential logarithmic single-valued neutrosophic hybrid average TODIM and hybrid geometric TODIM approaches, were the best choices of experiments. The robustness of the outcomes was adjudged using the sensitivity analysis approach, which has been carried out with respect to the criteria weights and the attenuation factor. The results show that the hybrid geometric TODIM approach produces more stable results, making it better than the hybrid averaging TODIM approach. The geometric TODIM rankings’ relative stability highlights its robustness and reliability, making it a stronger contender for sustainable EDM parameter selection.

Comparing different MCDM methods, like 2-tuple linguistic neutrosophic TODIM, Type-2 neutrosophic fuzzy TODIM, and exponential neutrosophic TODIM, shows that the ranking results are very consistent, with Experiment 2 coming out on top for most of them. Differences in the fuzzy sets used for linguistic evaluations account for minor discrepancies in rankings. Overall, the proposed approach shows distinct advantages in sustainability focused EDM decision-making, supporting a more effective, eco-friendly manufacturing process that aligns with the evolving standards of green manufacturing.

Future research could broaden the application of this framework beyond aluminum grade 5 to include other materials, such as titanium alloys and high-strength composites, which are essential in aerospace and biomedical sectors. This expansion would validate the adaptability of sustainable EDM practices across diverse materials and industries. Adding the suggested TODIM-based decision-support method to cutting-edge manufacturing technologies, like AI-driven process control and real-time monitoring, could also make the process more efficient and long-lasting. Further studies might also explore incorporating additional environmental metrics, such as carbon emissions and energy consumption, into the decision-making framework. This inclusion would allow for a more comprehensive evaluation of EDM’s environmental impact. Lastly, expanding on the linguistic evaluation methods with even more nuanced fuzzy sets, such as interval-valued or hesitant fuzzy logic, could provide a richer model for capturing expert uncertainty while enhancing the precision and robustness of sustainable EDM parameter optimization.

The computational complexity associated with the exponential logarithmic single-valued neutrosophic hybrid TODIM may pose a challenge in real-time applications, thereby limiting its practicality in dynamic manufacturing environments without further optimization. While environmental metrics are incorporated, the study does not include broader life cycle analysis, an aspect essential for complete sustainability assessments and can be carried out as a part of future research work.

## Electronic supplementary material

Below is the link to the electronic supplementary material.

Supplementary Material 1

## Data Availability

Data will be made available upon reasonable request to the corresponding author.
