# Supplementary material for: Optimization of process parameter for green die sinking electrical discharge machining: a novel hybrid decision-making approach
Source: Sci Rep. 2025 Apr 18;15:13489. doi: 10.1038/s41598-025-92713-2 (PMC12008234; doi:10.1038/s41598-025-92713-2)
Supplement: Supplementary file 1 — Supplementary Material 1 [file 41598_2025_92713_MOESM1_ESM.docx]

**Appendix A Summary of Recent Studies**

Table A1 Summary of Recent Studies on EDM Parameter Optimization Across Various Materials and Applications

| **Published study** | **Work material** | **Electrode and dielectric** | **Input variables** | **Output variables** | **Algorithm employed** |
| --- | --- | --- | --- | --- | --- |
| Seidi et al., [13] | Low Molybdenum alloy steel | Wire material was copper, distilled water was used as the dielectric fluid | wire feed speed, wire tension and generator power | Dimensional accuracy, hardness and roughness of product surface | Method based on the removal effects of criteria (MEREC) and weighted aggregates sum product assessment (WASPAS) |
| Varshney and Singh [14] | EN24 steel alloy | High-precision heavy-duty industrial keyless drill chuck | Gap current, pulse on time, rotational speed, and magnetic field assistance. | material removal rate, tool wear rate, overcut, and surface roughness | CRITIC (Criteria Importance Through Intercriteria Correlation)–TOPSIS |
| Nguyen et al., [15] | Titanium alloy | TiN coated tungsten carbide electrode | Voltage, capacitance, and the tool rotation | TWR and surface quality | Taguchi-Data Envelopment Analysis based Ranking |
| Tiwari and Panda [16] | Glass | Copper and nickel-coated copper coated tool | Voltage, tool feed rate, and machining time. | Radial overcut, circularity of the machined hole and heat-affected zone | Grey relational analysis (GRA), technique for order performance by similarity to ideal solution (TOPSIS), and VIšekriterijumsko KOmpromisno Rangiranje (VIKOR) |
| Mohankumar et al., [17] | Aluminum metal matrix composite | Material Brass and De-ionized water | Pulse ON time, Current, Voltage, and Pulse OFF time | Surface Roughness, Tool Wear Rate, and Material Removal Rate | Response Surface Methodology, hybrid method combining the Entropy Weight Method (EWM), Taguchi approach, TOPSIS, and GRA |
| Kavimani et al., [18] | Magnesium alloy (Mg–Li–Sr) | Brass coated copper wire electrode | Pulse OFF time, pulse ON time, wire feed rate, servo voltage and current | Kerf Width, Roughness of the surface, Material Removal Rate. | CRITIC -WASPAS multi-objective optimization method with Artificial Neural Network (ANN) |
| James et al., [19] | SS32750 super duplex stainless steels | Zinc-coated brass wire and De-ionized water | Pulse-on-time, current, and pulse-off-time | Material Removal Rate, surface roughness, and kerf width. | Evaluation based on Distance from Average Solution (EDAS) |
| Uyala et al., [20] | Magnesium alloy-ZE41 | Electrode material (copper, brass and EN8) | Electrode materials, peak current, pulse on time and pulse off time | Metal removal rate, tool wear rate, surface roughness, recast layer thickness and radial overcut | Measurement alternatives and ranking according to compromise solution (MARCOS), standard deviation (SD), Criteria importance through inter-criteria correlation (CRITIC), Method based on the removal effects of criteria (MEREC) |
| Pham Van et al., [21] | Ti-6Al-4V | Carbon-coated electrode and EDM oil | Voltage, spindle rotation, and capacitance | Z-coordinate depth of cut and tool wear rate | Deng’s similarity-based AHP method |
| Hadjela et al., [22] | AISI 4140 alloy steel | Coated carbide GC-2025 | Cutting speed, feed rate, and depth of cut | Mean roughness, flank wear and material removal rate | Taguchi, grey relational analysis (GRA), Technique by order of preference by similarity to ideal solution (TOPSIS), and multi-objective optimization ratio analysis (MOORA) |
| Abbas et al., [23] | Al/SiC/Gr composite | SS-304, copper, and brass | Pulse off time, pulse on time, servo voltage, current, and tool electrode | Material removal rate (MRR) and tool wear rate (TWR) | RSM–COPRAS |
| Sharma et al., [24] | Ti-6Al-4V |  | Pulse on-time: Pon; pulse off-time: Poff; servo voltage | Material removal rate, surface roughness, resultant cutting force, and noise level | Grey-Harmony search |
| Bhaskar et al., [25] | Ni50.3Ti29.7Hf20 alloy |  | Discharge time, Pause time, Gap voltage, and Wire travel speed | MRR and Surface roughness | TOPSIS |
| Danh et al., [26] | SKD11 tool steel | Copper electrodes | Powder concentration, the pulse current, the servo voltage, the pulse on time, and the pulse off time | Minimum surface roughness and maximum material removal speed | MAIRCA (Multi-Attributive Ideal-Real Comparative Analysis), MARCOS, TOPSIS and EAMR (Area-based Method of Ranking) |
| Pandiyan et al., [27] | AA6061-T6/15wt.% SiC composites | Electrolyte industrial copper electrode | Current, pulse on time, and gap voltage | Material removal rate, tool wear rate, circularity, and cylindricity | combinative distance-based assessment (CODAS) |
| Biswas et al., [28] | SS304 and SS316 materials | zinc-coated brass wire electrode | pulse on time, pulse off time, arc on time, arc off time, wire feed, and servo voltage | Material removal rate, kerf width, over cut, and surface roughness | TOPSIS |
| Phan et al., [29] | Titanium Alloy | Nickel Coated Aluminium Electrode | current, voltage and pulse on time | MRR and TWR | Preference Selection Index |
| Das et al., [30] | Ti–6Al–4V alloy | brass electrode | pulse-on time, peak current, gap voltage and flushing pressure | MRR, TWR, OC and taper | Weighted aggregated sum product assessment, technique for order of preference by similarity to ideal solution, combinative distance-based assessment and complex proportional assessment |
| Fuse et al., [31] | Ti6Al4V alloy | Molybdenum wire | Pulse-on time, pulse-off time, and current | Cutting speed, material removal rate, and surface roughness | Fuzzy analytic hierarchy process, TOPSIS |
| Zeng et al., [32] | Aluminum oxide | Electrolytic copper | Adhesive foil (type), peak current, Auxiliary current with high voltage, Pulse duration, Electrode jumping-up time, Servo reference voltage | Material removal rate, electrode wear rate, and surface roughness | Taguchi based TOPSIS coupled with AHP |
| Mandal and Mondal [33] | mild steel workpiece | Cu-MWCNT composite coated 6061Al electrode | pulse on time, duty cycle, discharge current, and gap voltage | EWR and MRR | TOPSIS |
| Shastri & Mohanty [34] | Nimonic C263 | copper, tungsten, and copper–tungsten electrodes | voltage, discharge current, pulse-on-time, duty factor, and electrode material | specific energy consumption, machining noise (N), material removal rate, electrode wear rate, surface roughness (Ra) and radial overcut (C) | preference ranking organization method for enrichment evaluation (PROMETHEE) |
| Yildiz [35] | Protherm C17500 Copper–Beryllium (Cu–Be) Alloy | Single hole copper tube electrode | Pulse-on/off time, duty cycle/factor and capacitance | Material removal rate and the electrode wear ratio | VIKOR, TOPSIS, GRA |
| Huu et al., [36] | SKD61, SKD11, SKT4 | Cu, Gr | Workpiece material, electrode material, electrode polarity, pulse on time, pulse off time, current, and powder concentration | material removal rate, tool wear rate, surface roughness, hardness surface, and white layer thickness | Taguchi–AHP–Deng’s method |
| Das and Chakraborty [37] | high carbon high chromium tool steel plate | copper rod | peak current, pulse duration, dielectric level and flushing pressure | process time, relative tool wear ratio, process energy, concentration of aerosol and dielectric consumption | superiority and inferiority ranking (SIR) method |
| Singh et al., [38] | Al-5083 alloy reinforced with B4C particles | Mo wire | Pulse on time, Pulse off time, induced current, wire feed, wt% of B4C. | average surface roughness, average peak to valley profile roughness, MRR, Kerf thickness | Fuzzy logic based multi-performance characteristics index |
